# Arthroscopically assisted conjoined tendon-coracoid tip complex transfer combined with Bankart repair without screws for traumatic anterior recurrent shoulder instability: clinical and imaging outcomes

**DOI:** 10.1016/j.jseint.2025.06.009

**Published:** 2025-07-08

**Authors:** Daisuke Mori, Noboru Funakoshi, Fumiharu Yamashita, Masahiko Kobayashi

**Affiliations:** a Department of Orthopaedic Surgery, Kyoto Shoulder & Sports Clinic, Kyoto, Japan; b Department of Orthopaedic Surgery, Kyoto Shimogamo Hospital, Kyoto, Japan

**Keywords:** Shoulder, Recurrent anterior instability, Cojoined tendon-coracoid tip transfer, Coracoid healing rates, Complications, Clinical outcomes

## Abstract

**Background:**

Screw-related complication rates after the Bristow–Latarjet procedure vary in studies. Hence, we utilized an arthroscopic-assisted conjoined tendon–coracoid tip complex transfer combined with Bankart repair without screws for anterior recurrent shoulder instability.

**Methods:**

This retrospective analysis of prospectively collected data included 51 shoulders in 48 patients (39 males, 9 females) who underwent the procedure with a mean age of 24.8 years (range, 15-69 years). Clinical assessments included the American Shoulder and Elbow Surgeons, Constant, Rowe, and Western Ontario Shoulder Instability Index scores. Postoperative complications, including recurrent instability (dislocation, subluxation, or positive apprehension sign), and revision surgeries, were documented. Computed tomography scans were utilized to evaluate postoperative graft healing status.

**Results:**

Clinical scores demonstrated significant improvement from preoperative levels to the final follow-up without revision surgery. Two patients (3.9%) experienced traumatic recurrent shoulder instability (subluxation) at 7 and 9 months postoperatively. At a mean follow-up of 26.7 months (range, 24 to 42), one of these patients reported satisfaction due to the absence of apprehension signs, whereas the other expressed mild satisfaction despite persistent apprehension. Forty-three (84.3%) coracoid grafts achieved complete healing with the scapular neck, whereas 4 (7.8%) exhibited partial healing, 3 (5.9%) showed resorption, and 1 (2.0%) experienced graft migration. Implant migration was not observed.

**Conclusion:**

The index procedure effectively improved shoulder stability and function, achieving a relatively high rate of coracoid graft union with no requirement for revision surgeries at a mean follow-up of 26.7 months.

The Bristow–Latarjet procedure is an increasingly utilized surgical approach for managing recurrent anterior shoulder instability, particularly among high-demand patients such as collision sports athletes or manual laborers.^5^^,^^28^^,^^31^^,^^46^ This procedure is associated with favorable clinical outcomes, typically rated as good to excellent. However, complication rates reported in the literature vary widely and include issues such as coracoid nonunion or osteolysis, screw displacement or breakage, and coracoid displacement.^1^^,^^6^^,^^8^^,^^11^^,^^17^^,^^20^^,^^22^^,^^36^ Of particular concern are screw-related complications, which may necessitate revision surgery because of impingement between the screw and the humeral head or because of the recurrent glenohumeral joint instability.^1^^,^^6^^,^^11^^,^^17^^,^^20^^,^^22^ Thus, the development of safer and more reliable surgical techniques is essential to minimize complications and the need for revision surgery, thereby enhancing clinical outcomes.

To address the challenges associated with screw-related complications in the surgical treatment of anterior shoulder instability, several screwless techniques have been proposed, although many have shown limited clinical success.^2^^,^^3^^,^^10^^,^^15^^,^^17^^,^^19^^,^^26^^,^^30^^,^^32^^,^^33^^,^^38^^,^^40^^,^^42^ Among these, Pascal et al introduced a technique using two cortical buttons on the anterior and posterior glenoid neck.^2^^,^^4^^,^^10^^,^^19^ However, this method involves releasing the coracoacromial (CA) ligament, which may result in anterosuperior shoulder translation in various joint configurations and loading conditions, regardless of intact rotator cuff function.^25^^,^^31^ Shao et al introduced an arthroscopic inlay Bristow using two cortical buttons. The method involves a 15 mm coracoid length osteotomy. One study showed that the mean distance from the coracoid tip to the anterior and posterior CA ligament was 7.8 mm and 25.7 mm, respectively.^14^ The inlay Bristow procedure results in the detachment of nearly 50% of the CA ligament. Therefore, techniques that mostly preserve the CA ligament are hypothesized to confer anterosuperior shoulder stability compared with the conventional coracoid transfer.

In pursuit of a technique that achieves favorable clinical outcomes without complications such as symptomatic impingement or the need for revision surgery as well as shoulder recurrent instability, we developed an arthroscopic-assisted conjoined tendon–coracoid tip complex (CTCTC) transfer combined with Bankart (capsular–labrum complex) repair. This approach keeps the CA ligament intact mostly and eliminates the need for coracoid fixation using metallic screws. The purpose of this study was to report the clinical and radiographic outcomes after this procedure.

## Materials and methods

The study protocol was approved by the local institutional review board, and informed consent was obtained from all patients before their inclusion in the study.

### Study population

This study was a retrospective review of prospectively collected data. A total of 55 patients with traumatic recurrent anterior shoulder instability underwent arthroscopic conjoined tendon transfer in conjunction with capsulolabral reconstruction between January 2018 and January 2022. The inclusion criteria were (1) traumatic recurrent shoulder instability with glenoid bone loss of <20%^5^^,^^12^^,^^45^ and (2) engagement in high-demand activities, defined as competitive or contact/forced overhead sports or occupations. Exclusion criteria included (1) previous shoulder surgery, (2) lost to follow-up within two years, (3) a diagnosis of epilepsy, or (4) pathological involvement of other soft tissues, such as the long head of the biceps or a rotator cuff tear.

### Surgical procedure and rehabilitation

**Figure 1:**
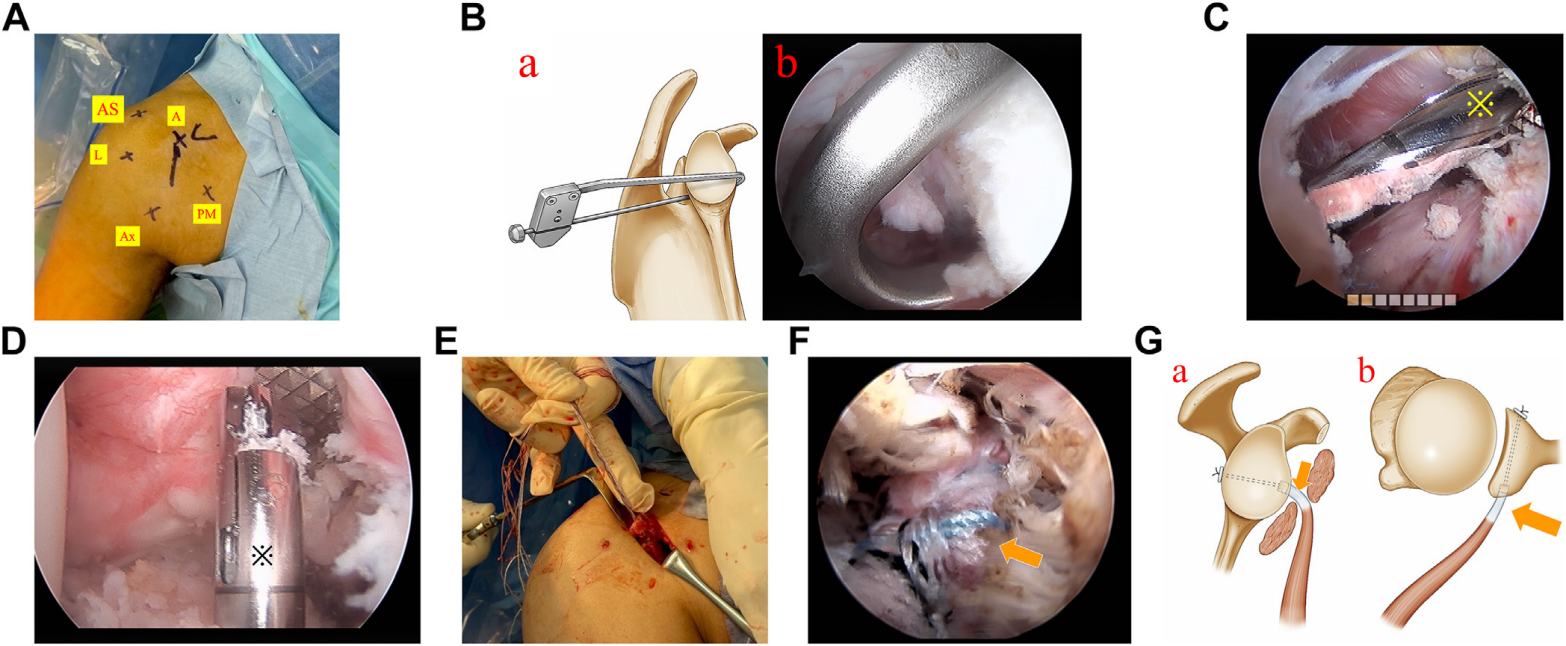
(**A**). The 5 anterior arthroscopic portals needed to perform an arthroscopically assisted coracoid tip–conjoined tendon transfer, as shown in the right shoulder of a patient in the beach-chair position: anterior (A), anterosupeior (AS), lateral (L), axially (Ax), and pectoralis major (PM) portals. The AS portal is mainly used for intra-articular work; it is located on the skin at the anterolateral corner of the acromion. The other 4 portals are mainly used to work extra-articularly. An anterior longitudinal incision, measuring 4-5 cm, is made laterally to the coracoid tip below the anterior portal for preparation of coracoid tip–conjoined tendon complex. (**B**) The glenoid guide (10° angulated) is introduced inside the shoulder with the help of a half-pipe cannula (a). The guide is positioned against the glenoid surface at the 4 o'clock position, with the tip of the hook located 10 mm medial to the glenoid rim (b, arthroscopic finding). (**C**) Being overdrilled with a 4.0-mm cannulated drill bit (※) to create a glenoid tunnel. (**D**) To create 8- or 8.5-mm anterior socket in the anterior glenoid neck at the 4-o'clock position using a 3.5-mm retrograde drill (※) for the right shoulder. (**E**) Coracoid tip and conjoined tendon complex. Mobilization of the coracoid tip through which 4 fiber-tapes are passed. (**F**) Observation of the transferred coracoid tip and conjoined tendon to which fiber-tapes (*arrow*) are sutured. (**G**) Fixing coracoid tip and conjoined tendon complex (*arrow*) into the glenoid neck after tying fiber tapes onto a suture button over the posterior orifice of the glenoid tunnel (a, en face glenoid; b, axial plane).

Highlights of the operative technique are shown in Video 1. The surgical procedure is performed with the patient positioned in the beach-chair configuration under general anesthesia. A posterior portal is established to facilitate the initial assessment of the glenohumeral joint. The first anterolateral portal is used for intra-articular work; it is located on the skin at the anterolateral corner of the acromion. Four additional anterior portals are created. The anterior (A) portal is 1 fingerbreadth lateral to the coracoid tip for preparation of Bankart repair; the axial (Ax) portal is located in the axillary fold, 2 to 3 fingerbreadths distal to the coracoid tip, and used for visualization during the coracoid preparation and subscapularis split; the lateral (L) portal is 2 fingerbreadths lateral to the A portal for visualization of coracoid and subscapularis split; and the pectoralis major muscle (PM) portal (passing obliquely through the PM) is 3 fingerbreadths medial to the Ax portal and used for soft tissue detachment of the medial coracoid, preparation of the subscapularis, and introduction of switch stick to protect the axillary nerve. Special care is paid to create the Ax and PM portals to avoid axillary nerve and vessel damage with use of electrocautery and the help of the spinal needle (Fig. 1, *A*).

A 30° arthroscope is introduced into the joint through the posterior portal. The labral-ligamentous complex is mobilized to the 6 o'clock position. A 7 o'clock (posteroinferior) portal is then established, followed by the creation of an anterosuperior portal as a secondary working portal. A suture anchor loaded with No. 2 high-strength sutures is inserted into the anteroinferior glenoid (at the 5:30 position) using a drill guide through the A portal. One suture limb from the anchor is placed through the inferior mobilized labrum via a passing suture (SutureLasso SD Wire Loop; Arthrex, Naples, FL, USA) using an intra-articular suture relay technique through the posteroinferior portal. The adjacent suture limb is similarly passed through the labrum. All anchor sutures are retrieved through the posteroinferior portal without knot-tying. A second suture anchor is then inserted at the 3 o'clock position. Sutures from the second anchor are retrieved through the anterosuperior portal.

The 70° arthroscope is introduced into the joint through the anterosuperior portal. Under direct visualization, a half-pipe cannula is advanced into the joint via the posterior portal. Subsequently, a customized glenoid drill guide is inserted through the posterior portal using the cannula as a conduit, which is then withdrawn from the joint. The guide is positioned against the glenoid surface at the 4 o'clock position, with the tip of the hook located 10 mm medial to the glenoid rim (Fig. 1, *B*). A 3-cm incision is made 1 cm medial and 1 cm inferior to the posterior portal site, and the outer sleeve is advanced onto the posterior glenoid neck. A 2.4-mm K-wire, housed within the outer sleeve, is drilled from posterior to anterior under guidance. The surgeon confirms that the K-wire exits the anterior glenoid neck at the 4 o'clock position (right shoulder) and 8 mm medial to the glenoid rim. Using switching sticks, the 70° arthroscope is transferred to the posterior portal. While a grasper holds the K-wire, it is overdrilled with a 4.0-mm cannulated drill bit to create a glenoid tunnel, exiting at the anterior glenoid neck, where it is secured with an arthroscopic rasp (Fig. 1, *C*). The 7-mm drill-sleeve (FlipCutter; Arthrex, Naples, FL, USA) is then tapped into the posterior glenoid neck through the K-wire, which is subsequently removed. A 3.5-mm retrograde drill is inserted into the glenoid tunnel in a non-flipped configuration via the drill-sleeve. The anterior portion of the glenoid tunnel is then enlarged to 8 or 8.5 mm to create an anterior socket, with a depth approximating 10 mm (Fig. 1, *D*).

The conjoined tendon and coracoid tip are exposed, and the CA ligament along with the pectoralis minor muscle is identified. Coracoid osteotomy is performed 8 mm from the coracoid tip. The CA ligament is minimally released at the osteotomy level, whereas the pectoralis minor is selectively released medially to a limited extent of 10-15 mm to avoid injury to the musculocutaneous nerve.^14^ The fascia surrounding the lateral, medial, and inferior aspects of the conjoined tendon is released digitally to ensure adequate mobility of the CTCTC.

An anterior longitudinal incision, measuring 4-5 cm, is made laterally to the coracoid tip below the anterior portal for the preparation of coracoid tip–conjoined tendon complex. The coracoid tip is stabilized using a Kocher clamp. A 2.4-mm K-wire is used to drill a hole through the middle of the coracoid tip, creating a proximal bony pillar approximately 8 mm long and 8 mm wide. Two No. 2 high-strength sutures (FiberTape; Arthrex) are braided around the conjoined tendon in a whipstitch configuration, extending 10 mm. The attached needle is passed through the drilled hole from the caudal to cranial direction and subsequently removed. A second hole is drilled in the coracoid tip, and an additional FiberTape is passed through in a similar fashion. This process results in four suture tapes penetrating the coracoid tip (Fig. 1, *E*). Then, the proximal part of the coracoid tip is fashioned with a rongeur to form a 5-mm-long, 8-mm-wide. The suture ends are retrieved via the PM portal. The anterior incision is then closed.

A straight blunt retractor, introduced through the PM portal, is used to protect the nerves. With the arm positioned by the side and slightly externally rotated, the subscapularis muscle is exposed and split horizontally using the half-pipe retractor. The spreader is gently advanced through the subscapularis muscle. The muscle belly is divided parallel to its fibers at the superior two-thirds and inferior one-third junctions. A cauterizing instrument is used to create a 1-cm longitudinal incision in the superficial tendon of the subscapularis. The half-pipe cannula is introduced via the PM portal for visualization of the anterior glenoid socket. The combination of the retractor and half-pipe cannula effectively establishes a safe passage through the subscapularis muscle.

A 2.4-mm cannulated guide pin is inserted from posterior to anterior via the drill-sleeve into the glenoid tunnel and exits through the subscapularis window. A passing suture (SutureLasso SD Wire Loop; Arthrex) is introduced through the guide pin and retrieved through the PM portal after removing the guide pin but before removing the drill-sleeve. Braided suture tapes attached to the coracoid-tip complex (CTCTC) are threaded through the passing suture at the PM portal, and the opposite end of the passing suture is pulled posteriorly, thereby drawing the tape ends through the subscapularis window and glenoid tunnel exiting through the drill-sleeve. The CTCTC is advanced through the subscapularis, with the coracoid pillar positioned within the glenoid socket. The drill-sleeve is then removed, and the surrounding soft tissue is cleared from the suture tapes manually. The two ends of one tape are passed separately through the middle two holes of a round button (Arthrex), whereas the two ends of the second tape are passed through the remaining middle holes. The button is advanced to the posterior orifice of the glenoid tunnel, where the suture ends are tied to their corresponding limbs on the button. This secures the CTCTC within the glenoid socket (Fig. 1, *F*) via suspension fixation with the round button positioned at the posterior orifice (Fig. 1, *G*).

Finally, the sutures attached to the capsulolabral complex from the anchor at the 5:30 position are tied. The previously placed suture anchor at the 3 o'clock position is used to repair the remaining capsulolabral complex. An additional suture anchor is placed at the 2 o'clock position to complete the repair of the capsulolabral complex.

### Postoperative care

Postoperative management involved immobilization of the patient's arm in a sling for a duration of four weeks. During this period, only passive shoulder motion within 90° of flexion and abduction was permitted. Active range-of-motion exercises commenced at five weeks postoperatively, whereas heavy lifting activities exceeding 10 pounds were restricted for the first 12 weeks. To minimize strain on the surgical repair, abduction and external rotation were avoided during the initial six weeks following surgery. Patients were cleared to resume all sports activities, including contact and overhead sports, within three to six months postoperatively, depending on their recovery trajectory based on the progression via physical therapy.

### Clinical assessment

Shoulder function and outcomes were evaluated using validated metrics, including the Constant score, the American Shoulder and Elbow Surgeons (ASES) score, the Rowe score, and the Western Ontario Shoulder Instability Index (WOSI). Assessments of active shoulder range of motion—specifically forward flexion, external rotation at the side, and both external and internal rotation at 90° of abduction—were performed preoperatively and at the final follow-up. Subjective evaluations of return to sports were quantified using the Single Assessment Numeric Evaluation, which expressed functional recovery as a percentage of the normal contralateral shoulder (0%-100%).^3^^,^^19^^,^^32^ Shoulder instability was assessed using the apprehension test. Data on postoperative recurrent instability, including dislocations, subluxations, or subjective instability confirmed by a positive apprehension test,^3^^,^^36^ were systematically recorded alongside any adverse events or revision surgeries.

### Structural assessment

**Figure 2:**
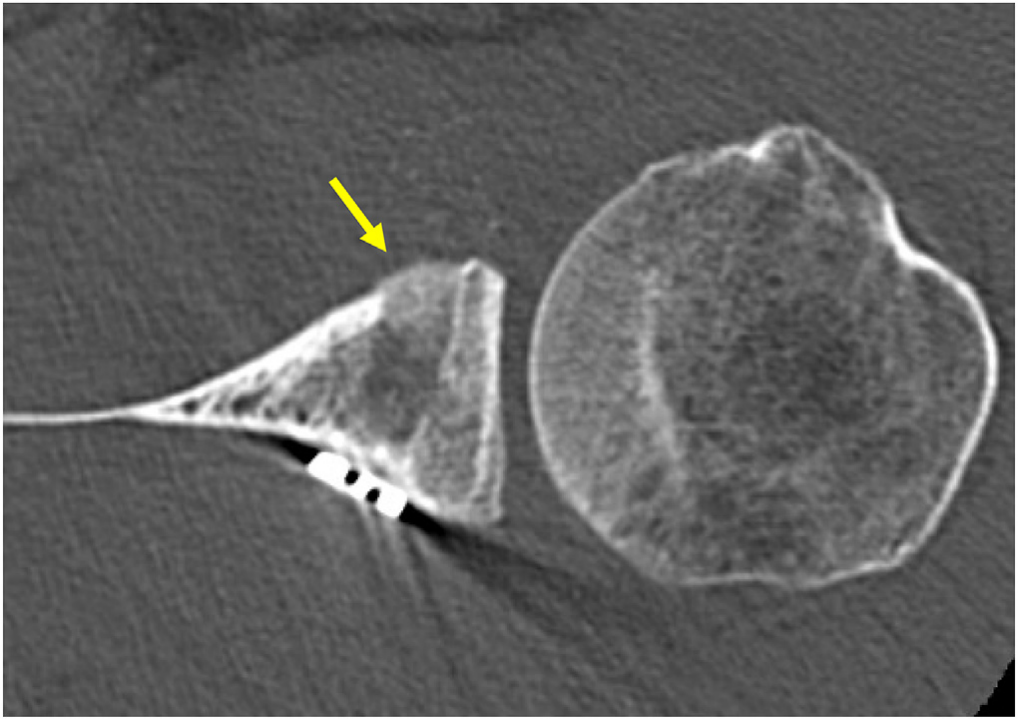
Complete graft healing. *Arrow*, the absence of a radiolucent line between the coracoid graft and the glenoid anterior rim.

**Figure 3:**
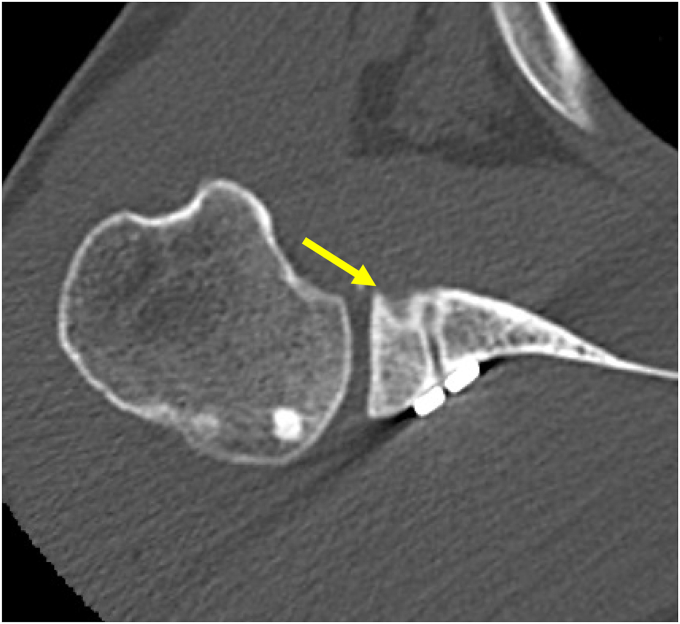
Partial graft healing. *Arrow*, a radiolucent line measuring <5 mm.

**Figure 4:**
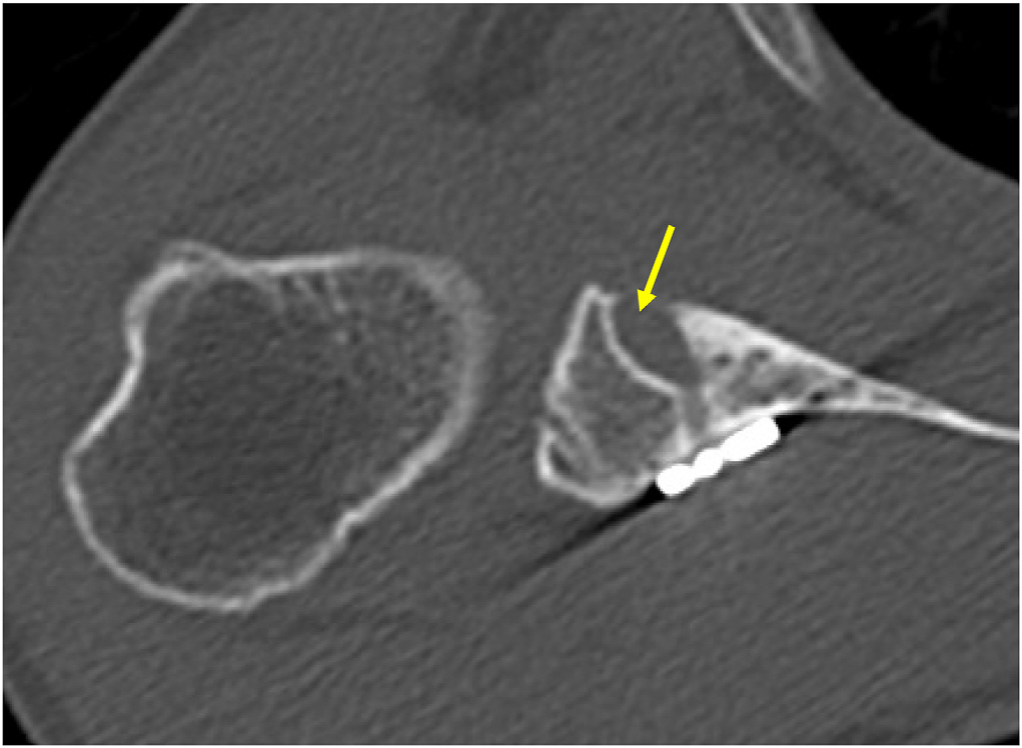
Graft resorption. *Arrow*, a radiolucent line >5 mm in the anterior glenoid socket.

**Figure 5:**
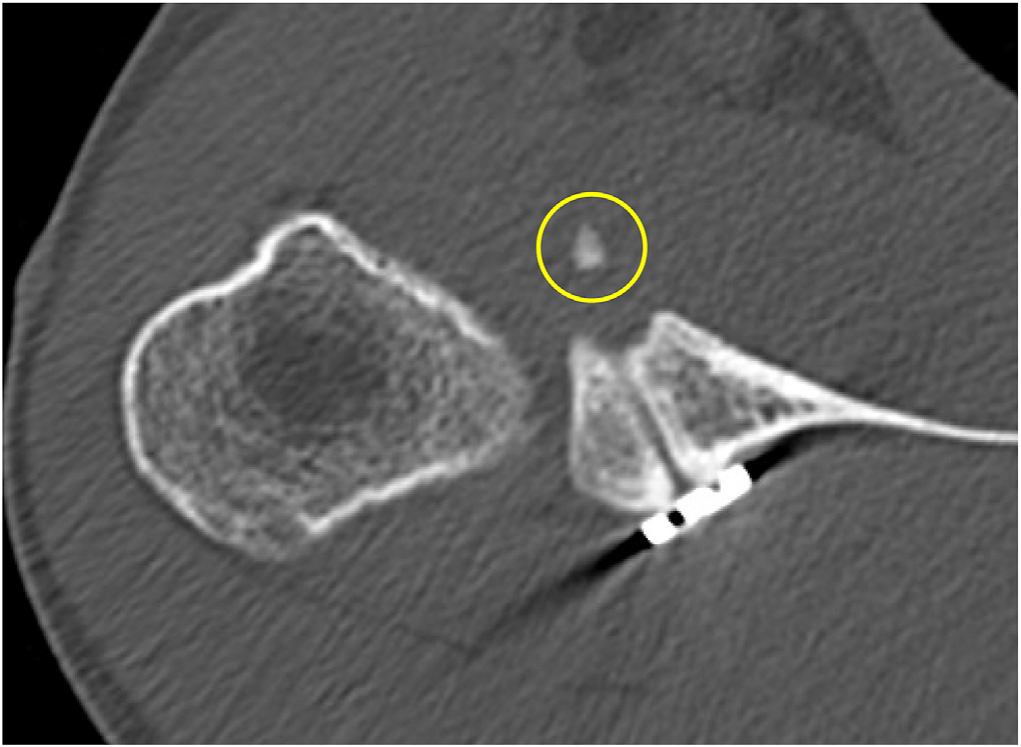
Graft displacement. Displacement of the graft () outside the glenoid socket.

Radiographic and computed tomography (CT) imaging were used for structural evaluations, including three-dimensional reconstructed CT scans obtained using an Activion 16 scanner (Toshiba, Tokyo, Japan) with the following parameters: spiral scanning, 0.5-mm slice thickness, pitch of 1, 0.4-mm reconstruction interval, and three-dimensional edit mode. The size of the glenoid bone loss was measured using a previously reported method.^35^ The Hill–Sachs lesion location was classified based on the on-/off-track concept lesions.^12^^,^^43^ Postoperative graft outcomes were categorized into four grades: (1) graft healing (Fig. 2), (2) partial healing (Fig. 3), (3) graft resorption (Fig. 4), and (4) graft migration (Fig. 5). Graft healing was defined by the absence of a radiolucent line between the coracoid graft and the glenoid anterior rim. Partial healing was indicated by a radiolucent line measuring <5 mm, whereas graft resorption was characterized by a radiolucent line >5 mm in the anterior glenoid socket.^26^^,^^32^ Graft migration was defined as the displacement of the graft outside the glenoid socket. Graft location was further evaluated, with the optimal bone block position defined as being located under the glenoid equator (between the 3 and 5 o'clock positions).^3^^,^^26^ A medialized coracoid graft was identified when the distance between the lateral edge of the glenoid socket and the glenoid rim exceeded 10 mm, consistent with the established criteria.^7^ Graft healing status and socket location was judged 1 year postoperatively. The diagnosis of graft healing was made by 1 treating surgeon and 1 blinded musculoskeletal radiologist who was not involved with this study and was blinded to the clinical findings. When there was a discrepancy between the assessments, the worse assessment was used. The diagnosis of socket location was made by the surgeon.

### Statistical analysis

Quantitative data were expressed as the mean ± standard deviation. Paired statistical testing with the Holm method was conducted to evaluate differences in the Constant, ASES, Rowe, and WOSI scores, as well as range of motion, between preoperative and postoperative assessments. Pearson's correlation coefficients were computed to explore associations between clinical variables—specifically the presence of dislocation or apprehension, postoperative WOSI scores, and postoperative Rowe scores—and factors such as the number of dislocations, patient age, involvement of the dominant hand, and radiographic graft classifications (eg, graft healing, graft partial healing, graft resorption, and graft migration). Stepwise linear regression analysis was used to identify independent variables influencing clinical outcomes. The kappa statistic, calculated using a two-way random-effects model, was applied to assess inter-rater reliability for graft healing status. Statistical significance was defined as *P* < .05. All analyses were conducted using Statistical Analysis System software (version 9.13; SAS Institute, Cary, NC, USA).

## Results

### Patient characteristics

From January 2018 to January 2022, 52 shoulders in 49 patients (40 males and 9 females) underwent arthroscopic conjoined tendon transfer with capsulolabral reconstruction. One male patient was lost to follow-up at 1 year postoperatively. Therefore, 51 shoulders in 48 patients (39 males and 9 females), with a mean age of 24.8 years (standard deviation, 10.2; range, 15-69 years), were included in the final analysis. The mean follow-up duration was 26.7 ± 3.8 (range, 24 to 42) months. Detailed demographic, medical history, and sports activity data are presented in Tables I and II.

**Table I:** Patients characteristics of cohort (n = 48∗).

Age (yr)†	24.8 ± 10.2
Dominant-side surgery (no. [%])	31 (60.1%)
Injured side (n), right/left	34/17
Follow-up period (mo)†	26.7 ± 3.8
No. of dislocations	5.2 ± 3.9
Glenoid bone loss (%) (range)	11.1 ± 4.0 (0-19.1)
0 (no. [%])	2 (3.9)
0-5 (no. [%])	0
5-10 (no. [%])	14 (27.5)
10-15 (no. [%])	29 (56.8)
15-20 (no. [%])	6 (11.8)
Off-track lesion (no. [%])	8 (15.7%)

∗ Cohorts involving 51 shoulders in 48 patients (3 patients undergoing for both shoulders).

† Values are given as the mean ± standard deviation.

**Table II:** Preoperative sports participation.

Rugby	20
Judo	6
American football	3
Bike	1
Tennis	1
Boxing	2
Wresting	1
Handball	1
Martial arts	1
Basketball	1
Baseball	1
Weights	2
None	4

### Clinical outcomes

At the final follow-up, significant improvements were observed in the ASES, Constant, Rowe, and WOSI scores (Table III, *P* < .001 for all). Notably, the mean improvement in the WOSI score exceeded the minimal clinically important difference of 220 across all shoulders. However, active forward flexion, external rotation, and internal rotation at 90° of abduction demonstrated significant reductions compared to preoperative values (*P* < .001).

**Table III:** Functional results preoperatively and at final follow-up.

	Preoperative	Postoperative	*P* value∗
ASES score			
Total	60.6 ± 23.9	97.9 ± 4.3	<.001
Pain	27.0 ± 17.2	49.0 ± 2.5	<.001
Function	34.0 ± 12.5	48.9 ± 2.4	<.001
Constant score			
Total	70.1 ± 13.2	93.2 ± 6.0	<.001
Pain	7.3 ± 5.2	13.8 ± 3.3	<.001
Strength	13.9 ± 6.1	19.6 ± 4.2	<.001
Rowe score			
Total	31.5 ± 6.4	97.7 ± 5.8	<.001
Function	18.8 ± 4.8	49.1 ± 3.6	<.001
Pain	2.8 ± 4.2/	9.2 ± 1.8	<.001
Stability	0.0 ± 0.0	29.4 ± 2.9	<.001
Mobility	10.0 ± 0.0	10.0 ± 0.0	-
SANE	-	94.7 ± 8.9	-
WOSI score			
Total	1386.4 ± 263.3	153.3 ± 170.5	<.001
Physical	589.9 ± 148.7	62.4 ± 84.9	<.001
Sports and recreation	285.4 ± 63.2	38.0 ± 35.8	<.001
Lifestyle	287.7 ± 65.2	27.3 ± 35.3	<.001
Emotions	223.8 ± 48.8	25.7 ± 24.1	<.001
ROM			
AE	176.0 ± 4.8	174.4 ± 4.5	<.001
ABD	171.1 ± 6.2	169.5 ± 5.8	.015
ER at the side	79.6 ± 4.8	77.8 ± 6.9	.014
ER at 90° of ABD	80.99 ± 7.9	78.1 ± 6.9	<.001
IR at 90° of ABD	81.5 ± 6.5	79.6 ± 6.5	<.001

*ASES*, American Shoulder and Elbow Surgeons; *SANE*, Single Assessment Numeric Evaluation; *WOSI*, Western Ontario Shoulder Instability Index; *ROM*, range of motion; *AE*, anterior elevation; *ABD*, abduction; *ER*, external rotation; *IR*, internal rotation.

Data are shown as mean ± standard deviation.

∗ Paired *t*-test with Holm methods.

### Return to sports and Single Assessment Numeric Evaluation

Among the 49 patients, 44 were engaged in sports activities before surgery. At the final follow-up, 95.5% (42/44) returned to sports at the same or higher level of performance compared with their preinjury status.

### Complications and revision surgery

Two patients (3.9%) experienced traumatic recurrent shoulder instability (subluxation) at 7 and 9 months postoperatively. At the final follow-up, one of these patients reported satisfaction due to the absence of apprehension signs, whereas the other expressed mild satisfaction despite persistent apprehension. None of them required revision surgeries. No cases of postoperative hematoma, infection, or injection for shoulder pain control, were observed. Transient musculocutaneous nerve palsy occurred in two patients, both of whom achieved full recovery within 15 weeks.

### Radiographic outcomes

Regarding graft positioning, the coracoid graft was located below the glenoid equator in 98.0% of shoulders (50/51) and at the 2-3 o'clock position in 1 shoulder (2.0%). No cases of excessive medial placement at the glenoid neck were identified. At the follow-up, complete graft healing to the scapular neck was observed in 43 shoulders (84.3%), partial healing in 4 shoulders (7.8%), and graft resorption in 3 shoulders (5.9%). Graft migration occurred in 1 case (2.0%). Posterior suture-button migration was not detected. Kappa analysis demonstrated substantial intraobserver reliability for graft status assessment, with a κ value of 0.648 (95% confidence interval, 0.423-0.874).

### Relationship between clinical variables and graft status

The association between clinical variables and graft status is summarized in Supplementary Table S1. The three graft categories (migration, resorption, and partial healing) demonstrated no significant correlation with clinical outcomes, including the presence of dislocation, apprehension, or postoperative WOSI and Rowe scores. However, the four graft categories (migration, resorption, partial healing, and complete healing) were significantly associated with the presence of apprehension (r = −0.313, *P* = .025). Notably, noncomplete healing grafts exhibited a negative correlation with apprehension (r = −0.350, *P* = .012). Stepwise linear regression analysis identified nongraft complete healing as the sole independent risk factor for the presence of apprehension (odds ratio, 0.071; 95% confidence interval, 0.006–0.913; *P* = .042). Despite this, no correlation was observed between clinical outcomes and graft healing status Supplementary Tables S2 and S3.

## Discussion

The present study demonstrates that the arthroscopically assisted CTCTC transfer combined with Bankart repair yields favorable clinical outcomes for patients with traumatic anterior shoulder instability. Notably, the mean improvement in the WOSI score exceeded the minimal clinically important difference of 220 across all shoulders.^30^^,^^27^ In addition, this procedure achieves a low recurrence rate and minimal complications while demonstrating graft healing rates comparable to previously reported studies on coracoid transfer with^6^^,^^20^^,^^31^^,^^34^^,^^36^^,^^37^^,^^39^ or without^3^^,^^10^^,^^19^^,^^26^^,^^33^ the use of screws, with a minimum follow-up of two years (Supplementary Table S4). The study reports an 84.3% rate of complete graft healing, accurate graft positioning below the equator in 98.0% of cases, and a 95.5% rate of return to sport at the same or higher level of performance without the need for revision surgeries. In addition, clinical outcomes—including scores and the presence of apprehension or instability—were not associated with graft healing status. However, noncomplete healing of the graft was significantly linked to the presence of apprehension sign, and it was identified as the only independent risk factor for this apprehension sign. These findings underscore the potential role of nongraft complete healing in contributing to positive apprehension sign. The clinical and radiographic outcomes observed in this study align well with those reported in the literature.^3^^,^^10^^,^^19^^,^^26^^,^^33^^,^^36^^,^^37^ Notably, the index procedure does not involve screw-related complications, suggesting it may serve as a viable alternative to conventional Bristow or Latarjet procedures, both with and without screws.

The conjoined tendon transfer comprises several steps aimed at minimizing complications such as nerve injury and coracoid malposition, even when the index procedure does not involve screw-related issues. Initially, preparation of the CTCTC is undertaken via a mini-open surgical approach, with meticulous attention paid to ensuring mobility and secure attachment of sutures or tapes to the CTCTC. Extensive literature highlights the risks associated with Bristow and Latarjet procedures, particularly concerning musculocutaneous and axillary nerve injury, with reported incidences ranging from 1.8% to 20.6%. Although some studies suggest that most nerve injuries following the Latarjet procedure are transient, others have documented cases of permanent axillary nerve weakness accompanied by sensory deficits.^6^^,^^9^^,^^16^^,^^24^ The authors recommended routine neurolysis to mitigate iatrogenic nerve damage, particularly during musculocutaneous nerve traction, findings that substantiate the necessity of releasing CTCTC during open surgery. Indeed, there were two patients with transient musculocutaneous nerve palsy in early series. Therefore, we pay special attention to the release of CTCTC during mini-open surgery based on the two cases and these studies.

The second critical step involves utilizing a glenoid guide to ensure accurate CTCTC positioning when creating a glenoid socket inferior to the equator. Typically, the coracoid graft is positioned at the 4 o'clock orientation for the right shoulder in the Bristow procedure and between 3 and 5 o'clock in the Latarjet procedure.^7^^,^^29^^,^^32^ Studies have demonstrated that neuroanatomic structures undergo positional alterations during the Bristow and Latarjet procedures.^9^^,^^16^ For instance, Freehill et al documented the medial displacement of the musculocutaneous and axillary nerves, resulting in potential vulnerability.^16^ Delaney et al identified the axillary and musculocutaneous nerves as being particularly at risk during the Latarjet procedure, especially during glenoid exposure and graft insertion.^9^ Based on these findings, introducing a glenoid guide can optimize creating of the anterior glenoid socket at the 4 o'clock orientation^18^^,^^21^ and facilitate transfer of the CTCTC through the subscapularis muscle tunnel, thereby potentially minimizing nerve-related complications.

Third, the index procedure incorporates a comprehensive capsulolabral repair (Bankart repair), in contrast to other conjoined tendon transfer techniques. This approach offers distinct advantages, including (1) maintaining the bumper effect and (2) preserving proprioceptive function.^2^^,^^4^^,^^3^^,^^10^^,^^19^^,^^36^^,^^39^ In a recent clinical study, Collin et al reported that persistent apprehension was observed in 30% of patients who underwent an isolated Latarjet procedure.^8^ Similarly, Boileau et al demonstrated the effectiveness of the combined Bristow–Latarjet procedure and Bankart repair, reporting favorable clinical outcomes and high rates of coracoid graft healing.^2^^,^^3^^,^^10^^,^^19^ These studies underscore the clinical utility of incorporating an additional capsulolabral repair in the procedure.^2^^,^^3^^,^^10^^,^^19^^,^^36^^,^^39^

The most notable aspect of the index procedure is the absence of metallic screws, which eliminates screw-related complications, such as concerns about screw backout, glenohumeral arthritis, or shoulder pain caused by prominent hardware. Clinical studies have demonstrated the efficacy of coracoid or conjoined tendon transfer without metallic screws in managing anterior glenohumeral instability.^2^^,^^3^^,^^10^^,^^15^^,^^19^^,^^26^^,^^30^^,^^32^^,^^33^^,^^38^^,^^40^ Boileau et al developed a comprehensive arthroscopic Latarjet procedure utilizing suture button fixation.^2^ Shao et al introduced the inlay Bristow technique, incorporating the mortise-and-tendon joint structure, which achieved superior clinical outcomes and high rates of coracoid graft healing.^32^ Our observed coracoid tip healing rates and clinical results align closely with the findings of these studies, thereby supporting the hypothesis of the present study.

Nevertheless, eight shoulders (15.7%) in the current study failed to achieve complete healing of the coracoid tip or graft. A possible explanation is that the coracoid tip is occasionally diminutive, resulting in a limited bone contact area with the glenoid socket, which may predispose to bone resorption.^32^^,^^34^^,^^42^ In addition, persistent instability could impede secure bone healing between the coracoid tip and the glenoid neck.^42^ However, among the two patients with recurrent instability (subluxation), one exhibited complete healing of the coracoid tip, whereas the other demonstrated incomplete healing. These observations suggest that anterior and inferior stabilization of the glenohumeral joint may depend more critically on the healing of the conjoined tendon to the glenoid neck than on coracoid tip healing.^13^^,^^15^^,^^23^^,^^41^^,^^44^ This hypothesis is corroborated by Douguih et al, who reported favorable clinical outcomes following conjoined tendon transfer without coracoid grafts.^15^ Furthermore, biomechanical investigations by Thomas et al revealed no significant differences between the Bristow procedure and conjoined tendon transfer alone in reducing anteroposterior translation in a simple tissue model without a bone defect.^41^ These findings imply that the sling effect of the conjoined tendon plays a more pivotal role in restoring anterior and inferior shoulder stabilization than coracoid healing in cases of glenoid bone defect <20%.^13^^,^^15^^,^^23^^,^^41^^,^^44^

However, incomplete graft healing was significantly associated with the presence of apprehension and was identified as the sole independent risk factor for the apprehension sign. On the other hand, Greco et al proposed that the presence of a medium or large Hill–Sachs lesion was the only risk factor significantly associated with persistent anterior apprehension.^19^ This discrepancy may be due to numbers of patients including Collision or Contact sports athletes, a medium or large Hill–Sachs lesion, graft healing process based on coracoid size (solid coracoid vs coracoid tip). Therefore, we could not definitely determine incomplete graft healing as the contraindication to clinical outcomes including persistent stability. The discrepancy, however, underscores the importance of ensuring complete coracoid graft healing to enhance the sling effect of the conjoined tendon as well as caution with patients with a medium or large Hill–Sachs lesion.

One superiority of the index procedure to other suture button fixation techniques was that removal of an 8-mm-long coracoid process keeps the CA ligament intact mostly, possibly leading to anterosuperior stability of the shoulder.^38^ One comparative study^34^ and one study by Tanaka et al^36^ proposed that the Latarjet procedure involved higher rates of subluxation and required more frequent injections for pain compared with the Bristow procedure. They discussed that the frequence was caused by possible anterosuperior instability following the amount of dissection of the CA ligament and pectoralis minor tendon.^34^^,^^36^ Indeed, the present study showed excellent pain relief (ASES and Constant pain scores, Table III) without injections after the index procedure as well as the return to sports rate (98.0%). These findings underscore the principle of the index procedure.

This study has several limitations. First, the small sample size and the absence of a control group—such as one undergoing open or arthroscopic Latarjet or conventional Bristow procedures—limit the generalizability of the findings.^34^^,^^36^ Second, the indication for the index procedure included glenoid bone loss of <20% based on the Sugaya method.^35^ The present study did not verify the effectiveness of the index procedure for shoulder with glenoid bone loss of ≥20%.^15^ Third, a biomechanical investigation is warranted to directly compare the index conjoined tendon transfer technique with established coracoid transfer methods, including the Latarjet and traditional Bristow techniques.^41^ The absence of implants on the anterior glenoid, however, reduces the risk of implant-related complications, such as nerve irritation, mechanical clicking, or persistent pain.^1^^,^^6^^,^^20^ Indeed, this study has demonstrated the clinical and radiographic efficacy of the index procedure, as evidenced by the absence of revision surgeries. These findings suggest that the index procedure may serve as a viable alternative to conventional coracoid transfer techniques that utilize screws. However, further studies are essential to more definitively establish the utility of the index procedure.

## Conclusion

This study represents the first series reporting clinical and radiographic outcomes after an arthroscopically assisted CTCTC transfer combined with Bankart repair without metallic screws for the treatment of traumatic anterior recurrent shoulder instability. The findings demonstrate a relatively high rate of graft healing, excellent graft positioning without revision surgeries at a mean follow-up of 26.7 months. These outcomes are comparable to those reported for conventional coracoid transfer with and without screws. Collectively, the results of this study support the potential of the index procedure as an effective alternative to conventional coracoid transfer techniques for managing recurrent shoulder instability.

## Acknowledgment

The authors thank Hajime Yamakage, M.D., for the statistical analyses, Yoshihiko Tsuda for medical illustrations (Davinci Medical Illustration Office), and Phoebe Chi, MD, from Edanz (https://jp.edanz.com/ac), for editing a draft of this manuscript.

## Disclaimers:

Funding: No funding was disclosed by the authors.

Conflicts of interest: The authors, their immediate families, and any research foundation with which they are affiliated have not received any financial payments or other benefits from any commercial entity related to the subject of this article.

## References

[1] Athwal G.S., Meislin R., Getz C., Weinstein D., Favorito P. (2016). Short-term complications of the arthroscopic Latarjet procedure: a North American experience. Arthroscopy.

[2] Boileau P., Gendre P., Baba M., Thélu C.É., Baring T., Gonzalez J.F. (2016). A guided surgical approach and novel fixation method for arthroscopic Latarjet. J Shoulder Elbow Surg.

[3] Boileau P., Saliken D., Gendre P., Seeto B.L., d'Ollonne T., Gonzalez J.F. (2019). Arthroscopic Latarjet: suture button fixation is a safe and reliable alternative to screw fixation. Arthroscopy.

[4] Boileau P., Thélu C.É., Mercier N., Ohl X., Houghton-Clemmey R., Carles M. (2014). Arthroscopic Bristow-Latarjet combined with Bankart repair restores shoulder stability in patients with glenoid bone loss. Clin Orthop Relat Res.

[5] Burkhart S.S., De Beer J.F., Barth J.R., Cresswell T., Roberts C., Richards D.P. (2007). Results of modified Latarjet reconstruction in patients with anteroinferior instability and significant bone loss. Arthroscopy.

[6] Butt U., Charalambous C.P. (2012). Complications associated with open coracoid transfer procedures for shoulder instability. J Shoulder Elbow Surg.

[7] Casabianca L., Gerometta A., Massein A., Khiami F., Rousseau R., Hardy A. (2016). Graft position and fusion rate following arthroscopic Latarjet. Knee Surg Sports Traumatol Arthrosc.

[8] Collin P., Rochcongar P., Thomazeau H. (2007). Treatment of chronic anterior shoulder instability using a coracoid bone block (Latarjet procedure): 74 cases. Rev Chir Orthop Reparatrice Appar Mot.

[9] Delaney R.A., Freehill M.T., Janfaza D.R., Vlassakov K.V., Higgins L.D., Warner J.J. (2014). 2014 Neer Award Paper: neuromonitoring the Latarjet procedure. J Shoulder Elbow Surg.

[10] Descamps J., Greco V., Chelli M., Boileau P. (2024). The arthroscopically guided Bristow-Latarjet procedure with cortical button fixation: a minimum 10-year follow-up. Am J Sports Med.

[11] Di Giacomo G., Costantini A., de Gasperis N. (2011). Coracoid graft osteolysis after the Latarjet procedure for anteroinferior shoulder instability: a computed tomography scan study of twenty-six patients. J Shoulder Elbow Surg.

[12] Di Giacomo G., Itoi E., Burkhart S.S. (2014). Evolving concept of bipolar bone loss and the Hill-Sachs lesion: from ‘‘engaging/non-engaging’’ lesion to ‘‘on-track/off-track’’ lesion. Arthroscopy.

[13] Dines J.S., Dodson C.C., McGarry M.H., Oh J.H., Altchek D.W., Lee T.Q. (2013). Contribution of osseous and muscular stabilizing effects with the Latarjet procedure for anterior instability without glenoid bone loss. J Shoulder Elbow Surg.

[14] Dolan C.M., Hariri S., Hart N.D., McAdams T.R. (2011). An anatomic study of the coracoid process as it relates to bone transfer procedures. J Shoulder Elbow Surg.

[15] Douoguih W.A., Goodwin D., Churchill R., Paulus M., Maxwell A. (2018). Conjoined tendon transfer for traumatic anterior glenohumeral instability in patients with large bony defects and anterior capsulolabral deficiency. Arthroscopy.

[16] Freehill M.T., Srikumaran U., Archer K.R., McFarland E.G., Petersen S.A. (2013). The Latarjet coracoid transfer procedure: alterations in the neurovascular structure. J Shoulder Elbow Surg.

[17] Garcia J.C., do Amaral F.M., Belchior R.J., de Carvalho L.Q., Markarian G.G., Montero E.F.S. (2019). Comparative systematic review of fixation methods of the coracoid and conjoined tendon in the anterior glenoid to treat anterior shoulder instability. Orthop J Sports Med.

[18] Gaujac N., Bouché P.A., Belas M., Bonnevialle N., Charousset C. (2024). The arthroscopic Latarjet procedure with a posterior guided system and suture-button fixation enables more precise bone block positioning in the axial plane versus anterior screws fixation. Knee Surg Sports Traumatol Arthrosc.

[19] Greco V., Descamps J., Catalan N.M., Chelli M., Joyce C.D., Boileau P. (2024). High rate of return to sport in contact and collision athletes after arthroscopic Latarjet with cortical button fixation. Am J Sports Med.

[20] Griesser M.J., Harris J.D., McCoy B.W., Hussain W.M., Jones M.H., Bishop J.Y. (2013). Complications and reoperations after Bristow-Latarjet shoulder stabilization: a systematic review. J Shoulder Elbow Surg.

[21] Kany J., Flamand O., Grimberg J., Guinand R., Croutzet P., Amaravathi R. (2016). Arthroscopic Latarjet procedure: is optimal positioning of the bone block and screws possible? A prospective computed tomography scan analysis. J Shoulder Elbow Surg.

[22] Kee Y.M., Kim J.Y., Kim H.J., Sinha S., Rhee Y.G. (2018). Fate of coracoid grafts after the Latarjet procedure: will be analogous to the original glenoid by remodelling. Knee Surg Sports Traumatol Arthrosc.

[23] Kephart C.J., Abdulian M.H., McGarry M.H., Tibone J.E., Lee T.Q. (2014). Biomechanical analysis of the modified Bristow procedure for anterior shoulder instability: is the bone block necessary?. J Shoulder Elbow Surg.

[24] Lafosse L., Lejeune E., Bouchard A., Kakuda C., Gobezie R., Kochhar T. (2007). The arthroscopic Latarjet procedure for the treatment of anterior shoulder instability. Arthroscopy.

[25] Lee T.Q., Black A.D., Tibone J.E., McMahon P.J. (2001). Release of the coracoacromial ligament can lead to glenohumeral laxity: a biomechanical study. J Shoulder Elbow Surg.

[26] Lin L., Zhang M., Song Q., Cheng X., Shao Z., Yan H. (2021). Cuistow: Chinese unique inlay Bristow: a novel arthroscopic surgical procedure for treatment of recurrent anterior shoulder instability with a minimum 3-year follow-up. J Bone Joint Surg Am.

[27] van der Linde J.A., van Kampen D.A., van Beers L.W.A.H., van Deurzen D.F.P., Saris D.B.F., Terwee C.B. (2017). The responsiveness and minimal important change of the Western Ontario shoulder instability index and Oxford shoulder instability score. J Orthop Sports Phys Ther.

[28] Mizuno N., Denard P.J., Raiss P., Melis B., Walch G. (2014). Long-term results of the Latarjet procedure for anterior instability of the shoulder. J Shoulder Elbow Surg.

[29] Nourissat G., Delaroche C., Bouillet B., Doursounian L., Aim F. (2014). Optimization of bone-block positioning in the Bristow-Latarjet procedure:a biomechanical study. Orthop Traumatol Surg Res.

[30] Patel V., Pearse E., Arnander M., Tennent D. (2021). Two-year results of arthroscopic conjoint tendon transfer procedure for the management of failed anterior stabilization of the shoulder. JSES Int.

[31] Schroder D.T., Provencher M.T., Mologne T.S., Muldoon M.P., Cox J.S. (2006). The modified Bristow procedure for anterior shoulder instability: 26-year outcomes in Naval Academy midshipmen. Am J Sports Med.

[32] Shao Z., Song Q., Cheng X., Luo H., Lin L., Zhao Y. (2020). An arthroscopic "inlay" Bristow procedure with suture button fixation for the treatment of recurrent anterior glenohumeral instability: 3-year follow-up. Am J Sports Med.

[33] Shao Z., Zhao Y., Luo H., Jiang Y., Song Q., Cheng X. (2022). Clinical and radiologic outcomes of all-arthroscopic Latarjet procedure with modified suture button fixation: excellent bone healing with a low complication rate. Arthroscopy.

[34] Song Q., Gao A., Bai J., Shao Z., Cui G. (2023). The arthroscopic Bristow procedure is superior to the arthroscopic Latarjet procedure in return to sports but inferior in graft healing: a comparative study with 3.4-year follow-up. Arthroscopy.

[35] Sugaya H., Moriishi J., Dohi M., Kon Y., Tsuchiya A. (2003). Glenoid rim morphology in recurrent anterior glenohumeral instability. J Bone Joint Surg Am.

[36] Tanaka M., Hanai H., Kotani Y., Kuratani K., Nakai H., Kinoshita S. (2022). Open Bristow versus open Latarjet for anterior shoulder instability in rugby players: radiological and clinical outcomes. Orthop J Sports Med.

[37] Tanaka M., Hirose T., Hanai H., Kotani Y., Kuratani K., Nakai H. (2024). Improvement of coracoid process union rates: a comparative study of conventional open and arthroscopic-assisted Bristow procedures for treating anterior shoulder instability in rugby players. J Shoulder Elbow Surg.

[38] Tang J., Zhao J. (2017). Arthroscopic transfer of the conjoined tendon-coracoid tip complex for anterior shoulder instability. Arthrosc Tech.

[39] Tasaki A., Morita W., Yamakawa A., Nozaki T., Kuroda T., Hoshikawa Y. (2015). Combined arthroscopic Bankart repair and coracoid process transfer to anterior glenoid for shoulder dislocation in rugby players: evaluation based on ability to perform sport-specific movements effectively. Arthroscopy.

[40] Tennent D., Colaço H., Arnander M., Pearse E. (2016). Arthroscopic conjoint tendon transfer: a technique for revision anterior shoulder stabilization. Arthrosc Tech.

[41] Thomas P.R., Parks B.G., Douoguih W.A. (2010). Anterior shoulder instability with Bristow procedure versus conjoined tendon transfer alone in a simple soft-tissue model. Arthroscopy.

[42] Xu J., Liu H., Lu W., Zhu W., Peng L., Ouyang K. (2019). Modified arthroscopic Latarjet procedure: suture-button fixation achieves excellent remodeling at 3-year follow-up. Am J Sports Med.

[43] Yamamoto N., Itoi E., Abe H., Minagawa H., Seki N., Shimada Y. (2007). Contact between the glenoid and the humeral head in abduction, external rotation, and horizontal extension: a new concept of glenoid track. J Shoulder Elbow Surg.

[44] Yamamoto N., Muraki T., An K.N., Sperling J.W., Cofield R.H., Itoi E. (2013). The stabilizing mechanism of the Latarjet procedure: a cadaveric study. J Bone Joint Surg Am.

[45] Zhu Y., Jiang C., Song G. (2017). Arthroscopic versus open Latarjet in the treatment of recurrent anterior shoulder dislocation with marked glenoid bone loss: a prospective comparative study. Am J Sports Med.

[46] Zimmermann S.M., Scheyerer M.J., Farshad M., Catanzaro S., Rahm S., Gerber C. (2016). Long-term restoration of anterior shoulder stability: a retrospective analysis of arthroscopic Bankart repair versus open Latarjet procedure. J Bone Joint Surg Am.

