# Supplementary material for: Arthroscopically assisted conjoined tendon-coracoid tip complex transfer combined with Bankart repair without screws for traumatic anterior recurrent shoulder instability: clinical and imaging outcomes
Source: JSES Int. 2025 Jul 8;9(6):1914–21. doi: 10.1016/j.jseint.2025.06.009 (PMC12828188; doi:10.1016/j.jseint.2025.06.009)
Supplement: Supplementary file 1 [file mmc1.docx]

Table S1. Correlation of clinical variables (presence of dislocation or apprehension, the postoperative WOSI and Rowe scores) and various variables such as dislocation times, age, and the involvement of dominant hand as well as radiographic graft various categories

	Graft_3 categories (n = 8)		Graft_4 categories (n = 51)		Graft_2 categories (n = 51)		Hill-Sachs (n = 51)		Dislocation times (n =51)		Dominant (n =51)		Old (n = 51)	
	ρ	P-value	ρ	P-value	ρ	P-value	ρ	P-value	ρ	P-value	ρ	P-value	ρ	P-value
Dislocation	0.361	.379	-0.168	.238	-0.191	.180	-0.087	.543	-0.007	.961	-0.045	.756	-0.317	.024
Apprehension	0.552	.156	-0.313	.025	-0.350	.012	-0.108	.451	-0.046	.747	-0.141	.325	-0.142	.320
Post_WOSI score_Total	0.496	.212	-0.016	.912	-0.040	.779	-0.256	.069	0.233	.100	-0.015	.917	0.159	.266
Post_Rowe score_Total	-0.309	.457	0.249	.078	0.265	.060	0.199	.162	0.028	.846	0.060	.678	-0.075	.601
Post_Constant score_Total	-0.100	.814	0.136	.342	0.141	.323	0.186	.192	0.012	.935	0.051	.721	-0.121	.399
Post_ASES score_Total	-0.160	.704	0.268	.057	0.275	.051	0.150	.292	-0.092	.520	-0.074	.608	-0.051	.720

Graft_3 categories involving variables; migration, absorption, and partial healing, Graft_4 categories involving variables; migration, absorption, partial healing, and complete healing. Graft_2 categories involving variables; non-complete healing (migrati and complete healing. Hill-Sachs categorial data involving varaiables on and off, Dislocation categorial data involving varaiables stable and subluxation, Apprehenison categorial data involving varaiables negative and positive.
