# Supplementary material for: Arthroscopically assisted conjoined tendon-coracoid tip complex transfer combined with Bankart repair without screws for traumatic anterior recurrent shoulder instability: clinical and imaging outcomes
Source: JSES Int. 2025 Jul 8;9(6):1914–21. doi: 10.1016/j.jseint.2025.06.009 (PMC12828188; doi:10.1016/j.jseint.2025.06.009)
Supplement: Supplementary file 2 [file mmc2.docx]

Supplementary Table S2 Odds Ratios for Postoperative Shouldres with subluxation Using Univariate and Multivate Logistic Regressions

	Univariate Analysis				Multivariate Analysis*			
	Odds	95% CI			Odds	95% CI		
	Ratio	Lower	Upper	*P* Value	Ratio	Lower	Upper	*P* Value
Graft_ complete Healing (vs. Non-complete healing)	0.167	0.009	2.984	.224	–	–	–	–
Graft_4 variables	0.651	0.131	3.226	.599	–	–	–	–
Dislocation times (per 1)	0.901	0.405	2.003	.798	–	–	–	–
Dominant_Yes (vs. No)	0.633	0.037	10.740	.752	–	–	–	–
Old (per 1)	0.247	0.051	1.206	.084	–	–	–	–

* From the results of the stepwise variable selection, any variables was not selected.

Graft non-complete healing involving variables; migration, absorption,, partial healing

Graft_4 variables involving variables; migration, absorption,, partial healing, and healing
