# Supplementary material for: Arthroscopically assisted conjoined tendon-coracoid tip complex transfer combined with Bankart repair without screws for traumatic anterior recurrent shoulder instability: clinical and imaging outcomes
Source: JSES Int. 2025 Jul 8;9(6):1914–21. doi: 10.1016/j.jseint.2025.06.009 (PMC12828188; doi:10.1016/j.jseint.2025.06.009)
Supplement: Supplementary file 3 [file mmc3.docx]

***Supplementary Table S3*** Odds Ratios for Postoperative Shouldres with apprehension Using Univariate and Multivate Logistic Regressions

| | Univariate Analysis | | | | Multivariate Analysis* | | | |
|---|---|---|---|---|---|---|---|---|
| | Odds Ratio | 95% CI Lower | 95% CI Upper | *P* Value | Odds Ratio | 95% CI Lower | 95% CI Upper | *P* Value |
| Graft_complete Healing (vs. Non-complete healing) | 0.071 | 0.006 | 0.913 | .042 | 0.071 | 0.006 | 0.913 | .042 |
| Graft_4 variables | 0.520 | 0.153 | 1.767 | .294 | – | – | – | – |
| Dislocation times (per 1) | 0.837 | 0.372 | 1.885 | .668 | – | – | – | – |
| Dominant_Yes (vs. No) | 0.300 | 0.025 | 3.549 | .340 | – | – | – | – |
| Old (per 1) | 0.991 | 0.876 | 1.121 | .886 | – | – | – | – |

* From the results of the stepwise variable selection, graft non-healed was selected.

Graft non-complete healing involving variables; migration, absorption,, partial healing

Graft_4 variables involving variables; migration, absorption,, partial healing, and complete healing
