# Supplementary material for: Arthroscopically assisted conjoined tendon-coracoid tip complex transfer combined with Bankart repair without screws for traumatic anterior recurrent shoulder instability: clinical and imaging outcomes
Source: JSES Int. 2025 Jul 8;9(6):1914–21. doi: 10.1016/j.jseint.2025.06.009 (PMC12828188; doi:10.1016/j.jseint.2025.06.009)
Supplement: Supplementary file 4 [file mmc4.docx]

Table S4 Comparative table with other results after coracoid transfer without screws based on clinical and radiographic outcomes including complications such as graft nonunion and revision srugery

| Author | Year | Technique | N | FU (mo) | Graft complete healing | Graft non-complete healing | Recurrent Instability | Revision surgery | Clinical Test | |
|---|---|---|---|---|---|---|---|---|---|---|
| Boileau et al[4] | 2019 | Suture-button Latarjet | 121 | 26.0 | 95.0% | nonunion (3.0%), migration (1.0%) | dislocation (0.8%) | 2.5% | Rowe | 90 |
| | | | | | | osteolysis (1.0%) | subluxation (2.5%) | | Walsh-Duplay | 91 |
| Greco et al[19] | 2024 | Suture-button Latarjet | 136 | 65.0 | 84.0% | nonunion (16.0%) | apprehension sign (26.0%) | 2.20% | Rowe | 91.0 |
| | | | | | | | dislocation (0.7%) | | Walsh-Duplay | 90.0 |
| Shao et al[31] | 2020 | Inlay Bristow | 56 | 36.1 | 96.4% | nonunion (3.6%) | subluxation (1.8%) | none | Rowe, ASES | 96.0, 92.5 |
| Lin et al[24] | 2021 | Inlay Bristow | 51 | 41.5 | 96.1% | nonunion (3.9%) | apprehension sign (2.0%) | none | Rowe, ASES | 96.0, 92.5 |
| Shao et al[32] | 2020 | Suture-button Latarjet | 30 | 38.0 | 96.7% | nonunion (3.3%) | no instabilty | none | Rowe, ASES | 95.9, 91.1 |
| Current study | 2025 | Conjoined tendon transfer | 51 | 26.7 | 84.3% | partial healing (7.8%), absorption (5.9%) | subluxation (3.9%) | none | Rowe, ASES | 97.7, 97.9 |
| | | | | | | migration (2.0%) | apprehension sign (2.0%) | | CS, WOSI | 93.2,153.3 |

N, included patients; FU, follow-up average in months; HI, horizontal instability; VI, vertical instability; CS, Constant score test (0-100)
